# Higher titers of some H5N1 and recent human H1N1 and H3N2 influenza viruses in Mv1 Lu vs. MDCK cells

**DOI:** 10.1186/1743-422X-8-66

**Published:** 2011-02-11

**Authors:** Sara B Hamilton, Diane E Wyatt, Brett T Wahlgren, Maureen K O'Dowd, Jane M Morrissey, Deirdre E Daniels, John A Lednicky

**Affiliations:** 1 Energy and Life Sciences Division, Midwest Research Institute, 425 Volker Boulevard, Kansas City, Missouri 64110, USA; 2 KC Bio, LLC, 2111 E. Santa Fe, Suite 312, Olathe, KS 66062, USA; 3 University of Kansas Medical Center, 3901 Rainbow Blvd. MS 1018, Kansas City, KS 66103, USA; 4 Environmental and Global Health Department, College of Public Health and Health Professions, University of Florida at Gainesville, Box 100188, Gainesville, FL 32610, USA

## Abstract

**Background:**

The infectivity of influenza A viruses can differ among the various primary cells and continuous cell lines used for such measurements. Over many years, we observed that all things equal, the cytopathic effects caused by influenza A subtype H1N1, H3N2, and H5N1 viruses were often detected earlier in a mink lung epithelial cell line (Mv1 Lu) than in MDCK cells. We asked whether virus yields as measured by the 50% tissue culture infectious dose and plaque forming titer also differed in MDCK and Mv1 Lu cells infected by the same influenza virus subtypes.

**Results:**

The 50% tissue culture infectious dose and plaque forming titer of many influenza A subtype H1N1, H3N2, and H5N1 viruses was higher in Mv1 Lu than in MDCK cells.

**Conclusions:**

The yields of influenza subtype H1N1, H3N2, and H5N1 viruses can be higher in Mv1 Lu cells than in MDCK cells.

## Background

The infectivity of influenza viruses can differ among the various primary cells and continuous cell lines used for such measurements [1,2]. As the term "infectivity" has many meanings in virology, in this manuscript, infectivity is broadly defined as the ability of a virus particle to enter a host cell and form viable progeny virions. Measures of infectivity depend not only on the inherent susceptibility of a particular type of cell for a given influenza virus, but also on the methodology used for infecting the cells [such as the length of time the virus is left in contact with the cells, as the affinity/avidity of a virus for its receptor(s) may vary according to cell type], the quasispecies distribution within a particular influenza virus stock, and other variables.

Accurate viable virus counts are essential for inhalation exposure studies with aerosolized viruses [3], for correlation of viable count to genome equivalence in level of detection studies, and other relevant work with influenza viruses. Quantitative RT-PCR methods are not suitable, as they do not distinguish between viable and non-viable virus particles. Indeed, infectious influenza virus particles comprise a minor subpopulation of biologically active particles (BAP) within a viral population [4]. The other BAP include interferon suppressing particles [4,5], defective interfering particles [4,6], and noninfectious cell-killing particles [4,7].

Madin-Darby canine kidney (MDCK) epithelial cells are widely used for the isolation of human influenza A and B viruses and the determination of influenza A virus titers [1,8-11]. However, we (S. Hamilton and J. Lednicky, unpublished) and others [2,12] have observed that all things equal, the cytopathic effects (CPE) of many influenza A viruses are detected earlier in a mink lung epithelial cell line (Mv1 Lu) (American Type Culture Collection [ATCC] CCL-64) than in MDCK cells. The use of Mv1 Lu cells for the detection of influenza viruses is not novel; for example, the cells are supplied by a commercial source (Diagnostic Hybrids, Inc., Athens, OH) to clinical laboratories for that purpose. In MDCK and Mv1 Lu cells grown as a monolayer, CPE due to influenza viruses generally consists of visible changes in the appearance of nuclei in infected cells, and the formation of focal enlarged granular cells or non-specific cell deterioration, followed by detachment of the swollen cells from the growing surface. Occasionally, influenza virus-infected Mv1 Lu cells form spindle-shaped granular cells that do not detach from the growing surface. A basic comparison of MDCK and Mv1 Lu cells is given in Table 1.

**Table 1:** Characteristics of MDCK and Mv1 Lu cells

Characteristic	MDCK	Mv1 Lu
Morphology	Epithelial	Epithelial

Growth properties	Adherent	Adherent

Source	Kidney	Lung

Gender	Female adult	Male and female from fetuses

Doubling time^1^	29 hr	20 hr

^1^Doubling time (this work): Time for cell to duplicate in DMEM under optimal conditions.

The acronym Mv1 Lu stems from "*Mustela vison *(American mink) lung" (now reclassified as *Neovison vison*). Mink are highly related to ferrets and are susceptible to influenza viruses [13]. We are performing various studies of influenza viruses in domesticated ferrets (*Mustela putorius furo*), and asked whether Mv1 Lu cells might be advantageous for the isolation and/or enumeration of H5N1 and other influenza viruses in ferret tissue specimens or secretions. An underlying assumption of ours was that influenza viruses in specimens derived from ferrets with active influenza infections would effectively attach, replicate and efficiently produce progeny virions in Mv1 Lu cells. Moreover, we wished to know whether virus yields might differ in MDCK vs Mv1 Lu cells. We learned that the virus yields of many low-passage influenza A virus strains was higher in Mv1 Lu cells than in MDCK cells, even when the virus had not been adapted for growth in ferrets.

## Results

### 1. Validation of cell lines

**Figure 1:**
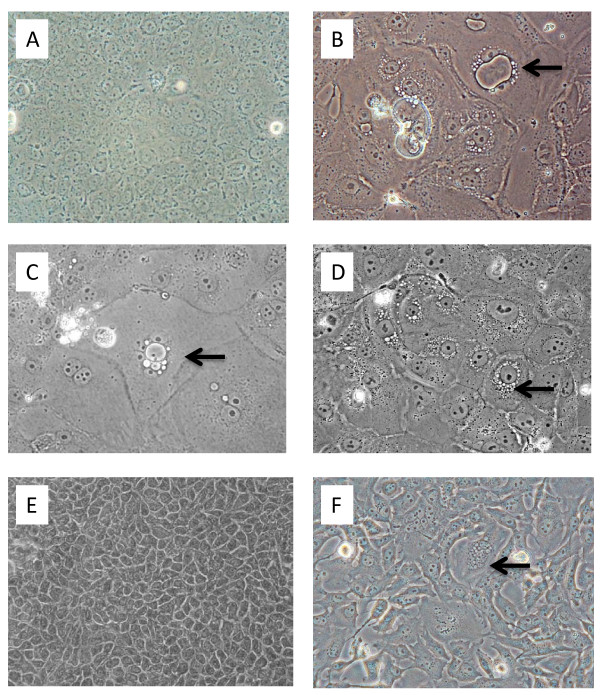
**Microscopic analyses of cultured MDCK and Mv1 Lu cells**. A. MDCK cells, normal (200× magnification); B. MDCK cells, aberrant; large inclusion body and vacuolation (arrow); C. MDCK cells, aberrant; Giant cell with inclusions (arrow); D. MDCK cells, aberrant; perinuclear vacuoles (arrow); E. Mv1 Lu cells, normal (200×); F. Mv1 Lu cells, aberrant; syncytium (arrow).

Whereas validated low-passage MDCK cells are used in some long-established influenza research laboratories, such cells are no longer easy to obtain. To gain insights applicable to current realities, MDCK and Mv1 Lu cells obtained from various commercial or university sources were evaluated for this work (the identity of most of the suppliers cannot be revealed due to legally binding client confidentiality agreements). The morphological characteristics of the MDCK and Mv1 Lu cells varied among the batches tested, and they also varied in sensitivity to influenza viruses, cell longevity, and cell growth kinetics/properties. Furthermore, especially since the cell lines were established long ago, they had been propagated by others in cell culture media supplemented with fetal bovine serum that had not been gamma-irradiated prior to cryopreservation/archiving. Not surprisingly, many batches of both cell lines contained numerous multinucleated large syncytia, cytoplasmic inclusion bodies, perinuclear or cytoplasmic vacuoles, and other signs of viral contamination, even in the presence of gamma-irradiated serum (some examples are shown in Figure 1).

**Figure 2:**
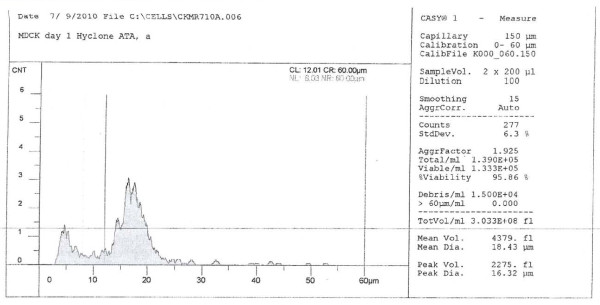
**MDCK cell profile under optimal conditions and serum, one day post-seed**. Ordinate, cell count (CNT); abscissa, diameter of cell or other particles (μm).

**Figure 3:**
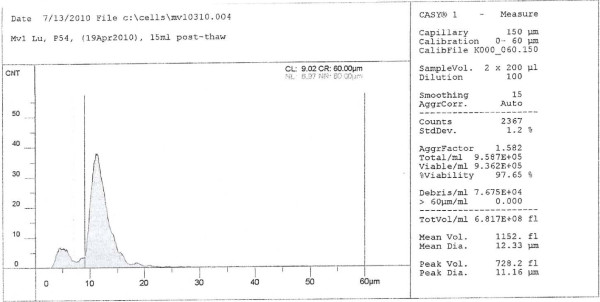
**Mv1 Lu cell profile under optimal conditions and serum, one day post-seed**. Ordinate, cell count (CNT); abscissa, diameter of cell or other particles (μm).

**Figure 4:**
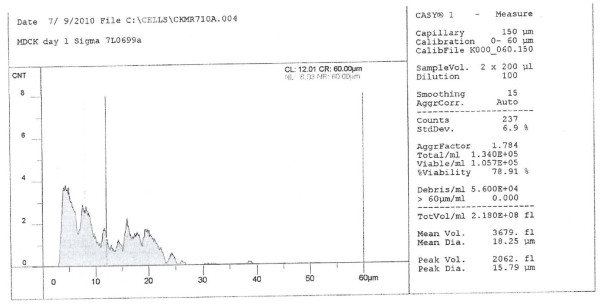
**MDCK cell profile under optimal conditions but with lower-quality serum, one day post-seed**. Ordinate, cell count (CNT); abscissa, diameter of cell or other particles (μm).

Various types of commercially prepared cell culture media and serum samples were extensively evaluated (data not shown). A batch each of MDCK and Mv1 Lu cells that lacked overt signs of viral (or other) contamination, and had minimal anomalies detectable by microscopy using phase-contrast objectives (no signs of non-specific cell deterioration, rare vacuoles and abnormal nuclei, no granulation, and less than 1 syncytium per 6 fields at 200× magnification), and that supported high-titer yields (≥ 4 × 10^8 ^PFU/ml) of *Influenza virus *A/Wisconsin/67/2005 (H3N2) (used as an indicator strain) in a series of pilot studies, were chosen for this work. During passage, the cells were sub-cultured at a minimum ratio of 1:4 to a maximum of 1:20 when they were nearly confluent. The dimensions of trypsinized MDCK and Mv1 Lu cells were measured using a CASY 1 resistance measuring multi-channel cell analyzer system (Roche Innovatis AG, Reutlingen, Germany). Those measurements indicated that the MDCK cells were larger than the Mv1 Lu cells. For example, one day post-seeding and cultivation under optimal conditions (Materials and Methods), MDCK had a mean diameter of 18.4 μm (Figure 2), whereas the mean diameter of Mv1 Lu cells was about 12.3 μm (Figure 3). As shown in Figure 2 and 3, MDCK cells consistently produced more debris than Mv1 Lu cells (compare areas of left peaks [level of debris] in both Figures). Cell dimension and amount of debris correlated with the source of both serum and growth media. For example, MDCK cells grown in optimal growth medium but sub-optimal serum had a different profile at 1 day post-seeding: the amount of debris was comparably higher and the cell dimension more variable (Figure 4).

### 2. Pandemic H1N1 2009 viruses can be propagated in Mv1 Lu cells

**Figure 5:**
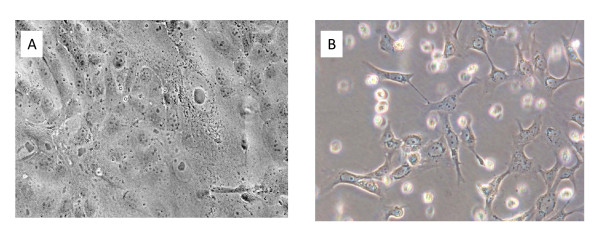
**Appearance of MDCK and Mv1 Lu cells 48 hr after infection of the cells at a high MOI with a pandemic H1N1 2009 virus**. Enlarged nuclei and cellular granulation but no cellular detachment are evident in the MDCK cells (panel A), whereas most of the Mv1 Lu cells have detached or are showing advanced CPE (panel B).

We determined that various pandemic H1N1 2009 viruses including strains A/California/04/2009 and A/California/07/2009 can be readily propagated in Mv1 Lu cells. As shown in Figure 5, A/California/04/2009, obtained for this work as an MDCK-passaged virus, is able to complete its replication cycle in Mv1 Lu cells. In Figure 5, MDCK and Mv1 Lu cells were infected at the same high multiplicity of infection (MOI) (approx 10); whereas some enlarged nuclei and cytoplasmic granulation (evidence of infection with influenza virus) are evident in the MDCK cells, most of the Mv1 Lu cells were destroyed 48 hrs post-infection. At a MOI of 0.1 or less, nearly complete destruction of the cells was delayed, requiring more than 48 hrs, but still following the previous pattern observed with high MOI: nearly complete destruction occurred first in monolayer of Mv1 Lu cells. Since the infected cells formed cytopathic effects (CPE) typical of influenza viruses at low and high MOI, the observed effects were presumed to be independent of effects attributable to those caused by non-infectious cell-killing virus particles. The virus in the Mv1 Lu cells (Figure 5) was putatively shown to be an influenza A virus using a commercial solid phase ELISA test (QuickVue Influenza A kit, Quidel Corp., San Diego, CA) kit, and confirmed as A/CA/04/2009 (H1N1) by RT-PCR and sequencing of the viral hemagglutinin, neuraminidase, and matrix genes (data not shown).

### 3. Mv1 Lu cells can be used for influenza A virus plaque assays

**Figure 6:**
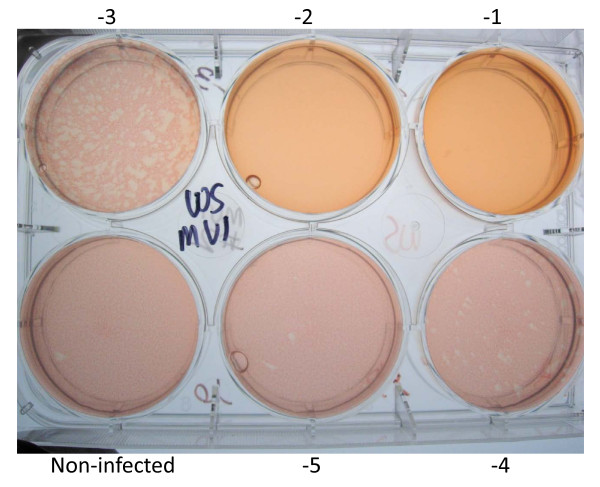
**Plaque assay of A/Mongolia/244/2005 (H5N1) in Mv1 Lu cells**.

**Figure 7:**
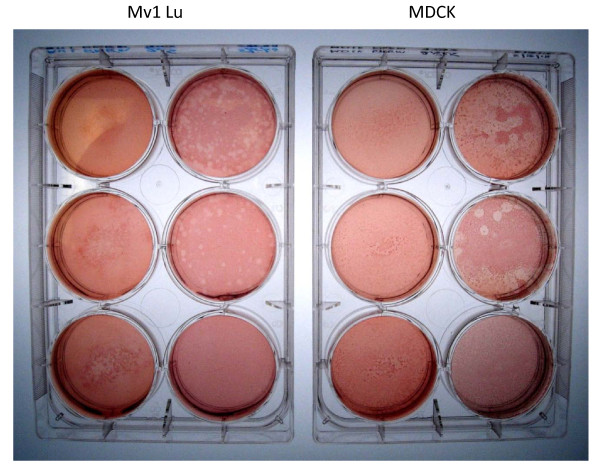
**Comparison of plaques formed by A/California/04/2009 (H1N1) in Mv1 Lu and MDCK cells**.

To our knowledge, Mv1 Lu cells have not been used for influenza virus plaque assays by other laboratories (supporting literature was not found). We now disclose that we have used Mv1 Lu cells for plaque assays of a variety of influenza A viruses. Examples are shown for A/Mongolia/244/2005 (H5N1) [Figure 6] and for A/California/04/2009 (H1N1) [Figure 7].

### 4. Titration of influenza H5N1 viruses

**Figure 8:**
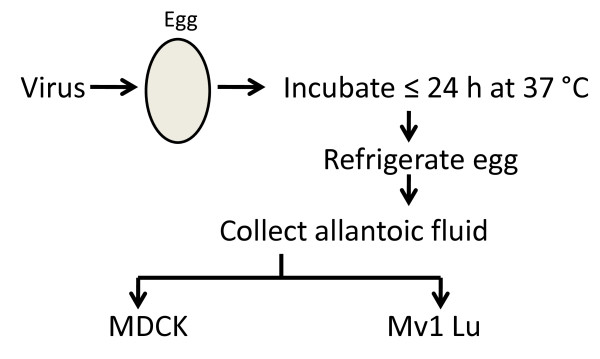
**Outline of process used for comparing titers of egg-grown virus in MDCK and Mv1 Lu cells**.

Following the general outline depicted in Figure 8, the plaque and 50% tissue culture infectious dose (TCID_50_) titers of 4 different H5N1 influenza viruses grown in embryonated chicken eggs were determined in MDCK and Mv1 Lu cells. The titers of the H5N1 viruses were higher in Mv1 Lu than MDCK cells (Table 2). For these H5N1 viruses, CPE were usually evident up to 6 hr earlier in Mv1 Lu cells than in MDCK cells (data not shown). Similar results were obtained with viruses first propagated in MDCK or Mv1 Lu cells instead of eggs as shown in Figure 8: higher titers resulted when Mv1 Lu cells were used for plaque or TCID_50 _determinations (data not shown).

**Table 2:** Titers of egg-grown H5N1 viruses in MDCK and Mv1 LU cells.

H5N1 Virus	MDCK cells		Mv1 Lu cells	
		
	Log_10_TCID_50_/ml	Log_10_PFU/ml	Log_10_TCID_50_/ml	Log_10_PFU/ml
A/Vietnam/1203/2004	7±0.2	6.8±0.2	8±0.2	7.9±0.2
A/Mongolia/244/2005	6.4±0.1	6±0.2	8±0.2	7.8±0.2
A/Iraq/207-NAMRU3/2006	8.8±0.2	8.3	10±0.2	9.9

*^a ^*TCID_50 _data from three separate experiments, plaque assay data from 1 (A/Iraq/207-NAMRU3/2006) or 3 (A/Vietnam/1203/2004 and A/Mongolia/244/2005) experiments.

### 5. Titration of seasonal and pandemic H1N1 2009 influenza viruses

Again following the procedure depicted in Figure 8, marginally to significantly higher viral titers were also calculated in Mv1 Lu cells than MDCK cells with various egg-grown seasonal and pandemic H1N1 2009 influenza viruses (Table 3). As for H5N1 viruses, similar results were obtained with viruses first propagated in MDCK and Mv1 Lu cells instead of eggs as shown in Figure 8: higher titers resulted when Mv1 Lu cells were used for plaque or TCID_50 _determinations (data not shown).

**Table 3:** Titers of egg-grown seasonal and 2009 pandemic influenza viruses in MDCK and Mv1 Lu cells^a^.

Virus	MDCK cells		Mv1 Lu cells	
		
	Log_10_TCID_50_/ml	Log_10_PFU/ml	Log_10_TCID_50_/ml	Log_10_PFU/ml
A/NWS/1933 (H1N1)	7.5±0.2	7.2±0.2	8.1±0.2	8±0.2
A/Puerto Rico/8/1934 (H1N1)	7.2±0.3	7.1±0.2	8.3±0.2	8.1±0.2
A/New Caledonia/20/1999 (H1N1)	8.1±0.2	ND^b^	8.5±0.2	ND
A/California/04/2009 (H1N1)	7.2±0.2	7±0.2	7.8±0.2	7.7±0.2
A/California/07/2009 (H1N1)	7.3±0.1	7.2±0.2	7.4±0.1	7.3±0.5
A/New York/18/2009 (H1N1)	7.9±0.2	ND	8.2±0.3	ND
A/Hong Kong/8/1968 (H3N2)	8.1±0.4	ND	8.8±0.2	ND
A/New York/55/2004 (H3N2)	7.8±0.2	ND	8.6±0.2	ND
A/Wisconsin/67/2005 (H3N2)	7.9±0.1	8.1±0.1	8.9±0.2	8.8±0.2

*^a ^*TCID_50 _and plaque assay data from three separate experiments.

^b^ND, not determined in these series of experiments.

## Discussion

As for MDCK cells, Mv1 Lu cells can be used for both influenza A virus TCID_50 _and plaque assays. And in establishing parameters for the enumeration of various influenza A strains, we observed that higher virus titers were attained for many in Mv1 Lu cells compared to the titer obtained in the more commonly used MDCK cell line. Taken together, it may be advantageous to use Mv1 Lu cells for certain applications, such as for obtaining estimates of a delivered dose of aerosolized influenza virus in inhalation exposure studies [3], and for the detection of relatively small amounts of infectious influenza virus particles in tissue or secretion specimens.

Though not specifically mentioned in the body of this manuscript, we noted that Mv1 Lu cells must be handled as described (Materials and Methods). When Mv1 Lu cells are maintained confluent for more than 1 week without splitting them for virus titration, variability in the CPE caused by influenza viruses occurs, as reported by Schultz-Cherry *et al *[2]. In the improperly maintained Mv1 Lu cells, the appearance of CPE is typically delayed compared to MDCK cells, and these Mv1 Lu cells cannot be used for plaque assays.

## Materials and methods

### 1. Cells

MDCK and Mv1 Lu from Diagnostic Hybrids, Inc. were selected for this work. The MDCK cells were obtained at passage 68 and used through passage 78, Mv1 Lu cells at passage 71 and used through passage 88. Both cell lines were cultured as monolayers at 37ºC and 5% CO_2. _Both cell lines were routinely propagated in either Dulbecco's Modifed Eagle's Medium (DMEM) (Mediatech, Inc., Manassas, VA) or Earle's Minimal Essential Medium (EMEM) (Invitrogen Corp., Carlsbad, CA). Both medium formulations were supplemented with 2 mM L-Alanyl-L-Glutamine (GlutaMAX™, Invitrogen Corp.), antibiotics [PSN; 50μg/ml penicillin, 50 μg/ml streptomycin, 100 μg/ml neomycin (Invitrogen Corp.)], and 10% (v/v) heat-inactivated gamma-irradiated fetal bovine serum (HyClone, Logan, Utah). Sodium pyruvate (Invitrogen Corp.) and non-essential amino acids (Hyclone) were added to EMEM (each at a manufacturer's recommended final concentration of 1×). Prior to virology tests, the following precautionary steps were taken to reduce possible mycoplasma and other contamination: the pre-banked cells were propagated in plasmocin (Invivogen, San Diego, CA) containing growth media for two weeks, followed by two weeks in the absence of antibiotics to determine whether fast-growing microbial contaminants were present or abnormal morphological changes would occur. The cells were confirmed negative by PCR for the presence of mycoplasma DNA using a Takara PCR Mycoplasma Detection kit (Takara Bio, USA, Thermo Fisher), and by mycoplasma tests (culture and DNA staining) performed by a commercial testing laboratory (Bionique Testing Laboratories, Saranac Lake, NY). The cell lines were observed daily to monitor confluency and checked for normal morphology. They were split when they reached ~90% confluency. Cell counts were performed on harvested cultures using either trypan blue dye-exclusion hemacytometer methodology or by automated cell size analysis (CASY 1 counter).

Actively growing MDCK and Mv1 Lu cells were planted at 1-3 × 10^3 ^viable cells/well in 96 well microtiter plates a minimum of three days prior to assay. Likewise, 6-well microtiter plates were planted at least three days prior to performing a plaque assay at a seeding density of 3-6 × 10^5 ^viable cell/well. Cell banks were prepared by freezing actively growing cells in standard growth medium containing 5% DMSO. After rapid thawing of frozen vials of cells, each vial was centrifuged, the freeze medium was discarded, and the cell pellet was resuspended in growth medium for planting culture vessels.

### 2. Influenza viruses

Influenza virus H5N1 strains A/Vietnam/1203/2004 and A/Mongolia/244/2005 were from archives of the Southeast Poultry Research Laboratory, and A/Iraq/207-NAMRU3/2006 was from the National Biodefense Analysis and Countermeasures Center [NBACC], which obtained the virus from Naval Medical Research Unit No. 3 [NAMRU-3], Cairo, Egypt (37) (Table 1). The H5N1 viruses were received as low-passage stocks, and their identity verified using viral genomic sequencing. Other influenza viruses were obtained from the Centers for Disease Control and Prevention or from the ATCC.

### 3. Virus propagation

Low-passage stocks of the H5N1 viruses were propagated in the allantoic cavities of 10-day-old embryonated chicken eggs (ECE) for 24 h at 37°C; the allantoic fluid was harvested, centrifuged for clarification, and stored at -80°C for up to one year or in the vapor phase of liquid nitrogen for longer storage [3,11,14,15]. All work with H5N1 viruses was conducted in a Biosafety Level 3 enhanced laboratory. Subtype H1 and H3 influenza viruses were propagated in ECE or in MDCK cells (using the same substrate they were propagated in at the ATCC or CDC).

### 4. Calculation of TCID_50 _values

TCID_50 _values were determined by infecting MDCK and Mv1 Lu cells in 96-well microtiter plates with serial dilutions of virus and calculation of the TCID_50 _four days later by the method of Reed and Muench [16]. Cells for TCID50 determinations were in serum-free complete EMEM containing L-1-tosylamide-2-phenylethyl chloromethyl ketone (TPCK)-treated trypsin, and re-fed with the same medium. As the specific activity of the TPCK-trypsin was relatively high, the final concentrations of TPCK-trypsin were 1.0 μg/ml for MDCK cells and 0.1 μg/ml for Mv1 Lu cells.

### 5. Plaque assays

For plaque assays, newly confluent MDCK or Mv1 Lu cells in six-well tissue culture plates were inoculated with 0.2 ml of virus serially diluted in serum-free complete EMEM containing TPCK-treated trypsin. Virus was adsorbed to cells for 1 hr at 37ºC (H5N1) or 35 ºC (seasonal influenza viruses) with rocking every 15 min (2009 pandemic influenza H1N1 were adsorbed for 2 h at 35 ºC). After virus adsorption, the cells were washed with serum-free EMEM and the wells overlaid with 3 ml/well of primary overlay consisting of 1.6% w/v agarose (Invitrogen Corp.) mixed 1:1 with serum-free 2X EMEM (Lonza, Walkersville, MD) containing antibiotics and TPCK-trypsin. With the exception of H1N1 pandemic influenza 2009 viruses, plates were inverted and incubated for 3 days at temperatures appropriate for the viruses, then overlaid with 2 ml of secondary overlay of 1.6% w/v agarose mixed 1:1 with 2X EMEM containing 0.14 mg/ml neutral red (catalog number N2889, Sigma-Aldrich, St. Louis, MO), the plates inverted, and incubated for two additional days to visualize plaques. The pandemic 2009 H1N1 viruses required longer incubation times: 3 days after the primary overlay (performed as described above), the cells were overlaid 2 ml/well of 1.6% w/v agarose (Invitrogen Corp.) mixed 1:1 with serum-free 2X EMEM (Lonza) containing antibiotics and TPCK-trypsin, inverted, and incubated for another 2 days. They were subsequently overlaid with 2 ml of secondary overlay with neutral red as described above, and the plates incubated for 2 additional days to visualize plaques.

## Competing interests

The authors declare that they have no competing interests.

## Authors' contributions

SBH established accurate virus quantification procedures, performed plaque and TCID_50 _assays, assisted with data interpretation, propagated MDCK and Mv1 Lu cells, provided photographs related to cell morphology and plaque assays, performed trypsin dose response analyses, engaged in trouble-shooting and cell-banking, and helped write the manuscript; DEW performed plaque and TCID_50 _assays, assisted with data interpretation, propagated MDCK and Mv1 Lu cells, engaged in trouble-shooting and cell-banking, established cell-growth curves, performed plasmocin-related procedures, mycoplasma tests, CASY analyses, cell banking, provided figures related to CASY analyses, and helped write the manuscript; BTW and MKO assisted with plaque and TCID_50 _assays, propagated MDCK and Mv1 Lu cells, engaged in cell-banking, established cell-growth curves, performed plasmocin-related procedures, mycoplasma tests, and CASY analyses; JMM propagated MDCK and Mv1 Lu cells, engaged in cell-banking, established cell-growth curves, and established many record-keeping and monitoring protocols; DED performed plaque and TCID_50 _assays, assisted with data interpretation, performed trypsin dose response analyses, engaged in trouble-shooting, and managed most programs relevant to the work of this manuscript; JAL conceived of the use of Mv1 Lu cells, was involved in most aspects of this work (excluding CASY analyses and plasmocin treatments) and co-prepared the manuscript. All authors read and approved the final manuscript.
